# Poly (ADP-ribose) polymerase 1 transcriptional regulation: A novel crosstalk between histone modification H3K9ac and ETS1 motif hypomethylation in BRCA1-mutated ovarian cancer

**DOI:** 10.18632/oncotarget.1549

**Published:** 2013-12-29

**Authors:** Da Li, Fang-Fang Bi, Ji-Min Cao, Chen Cao, Chun-Yan Li, Bo Liu, Qing Yang

**Affiliations:** ^1^ Department of Obstetrics and Gynecology, Shengjing Hospital of China Medical University, Shenyang, China; ^2^ Department of Physiology and Pathophysiology, Institute of Basic Medical Sciences, Chinese Academy of Medical Sciences, School of Basic Medicine Peking Union Medical College, Beijing, China; ^3^ Department of Pathology, Chinese PLA General Hospital, Beijing, China; ^4^ Department of Histology and Embryology, Institute of Basic Medical Sciences, Chinese Academy of Medical Sciences, School of Basic Medicine Peking Union Medical College, Beijing, China; ^5^ Department of Laboratory Medicine, No. 1 Hospital of China Medical University, Shenyang, China

**Keywords:** PARP1, H3K9ac, ETS1, BRCA1, Ovarian cancer

## Abstract

Poly (ADP-ribose) polymerase 1 (PARP1) plays a critical role in ovarian cancer progression. However, the epigenetic mechanism regulating PARP1 transcription remains largely unknown. Here, we show that the hypomethylated ETS1 motif is a key regulatory element for the PARP1 gene in BRCA1-mutated ovarian cancer. Mechanistically, the ETS1 motif hypomethylation-mediated increase of active histone marker H3K9ac and transcription factor ETS1 enrichment synergistically activates PARP1 transcription. Clinicopathological data indicate that a hypomethylated ETS1 motif was associated with high-grade tumors (*P* = 0.026) and pN1 (*P* = 0.002). Univariate survival analysis demonstrated an association between the hypomethylated ETS1 motif and an increased risk of death in BRCA1-mutated ovarian cancer patients. Our findings imply that the genetic (such as BRCA1 mutation) and epigenetic mechanisms (such as hypomethylated ETS1 motif, and histone modification H3K9ac and transcription factor ETS1 binding) are jointly involved in the malignant progression of PARP1-related ovarian cancer.

## INTRODUCTION

Ovarian cancer is characterized by a high mortality rate among gynecologic malignancy worldwide [1]. Accumulating evidence indicates that hereditary factors (e.g., BRCA1) [2] and epigenetic events [3,4] are involved in the initiation and progression of ovarian cancer. However, the joint effect of genetic and epigenetic mechanisms has not been extensively studied. In 2005, two pivotal studies showed that BRCA-deficient cells were especially sensitive to chemical inhibitors of poly (ADP-ribose) polymerase (PARP), which plays a critical role in single-stranded DNA break repair, presumably due to the lack of homologous recombination-dependent DNA repair [5,6]. These findings have raised significant concerns about PARP in BRCA-deficient ovarian cancer.

Our previous results suggested that hypomethylation of the promoter region, especially around the ETS1 motif may be responsible for PARP1 overexpression in BRCA1-mutated serous ovarian cancer [7]. However, the exact mechanism of abnormal promoter methylation-related PARP1 expression is still not entirely clear. Therefore, the present study was undertaken to investigate PARP1 transcriptional regulation from genetic (BRCA1 mutation) and epigenetic (promoter methylation, histone modifications and transcription factor binding) aspects, and to provide novel insight into epigenetic change-mediated abnormal PARP1 expression in BRCA1-mutated ovarian cancer progression.

## RESULTS

### Hypomethylated ETS1 motif is a key regulatory mechanism for PARP1 transcription in BRCA1-mutated ovarian cancer

**Figure 1:**
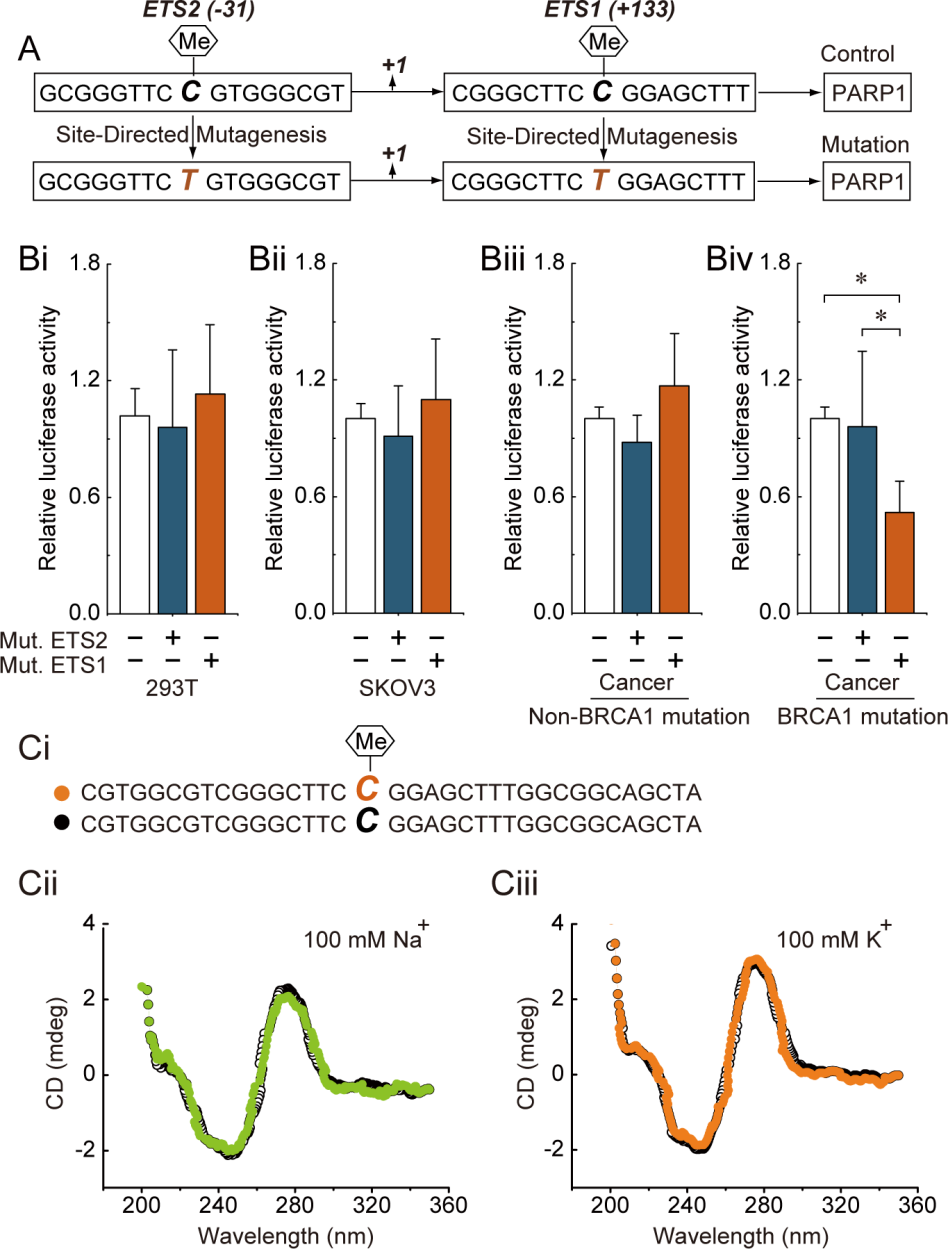
ETS1 motif methylation and PARP1 transcriptional activity (A) the schematic represents the cytosine located at the ETS1 and ETS2 motif that were point mutated to generate the thymine. (Bi-Biv) 293T cells, SKOV3 cells and primary non-mutated and BRCA1-mutated ovarian cancer cells were transfected with mutant plasmids. At 24 hours after transfection, whole-cell extracts were analyzed for luciferase activity. Bar graphs show mean ± SD. *P < 0.05 vs. Control. Mut., Mutation. (Ci) the schematic represents the selected nucleotide sequence with or without a methyl group at the fifth position of the cytosine pyrimidine ring at the ETS1 motif. (Cii) the CD spectra of the selected nucleotide sequence in the presence of 100 mM Na+ or 100 mM K+ are shown.

Our previous results suggested that promoter hypomethylation, especially around the ETS1 and ETS2 motifs, may be involved in the up-regulation of PARP1 expression in BRCA1-mutated ovarian cancer. To further confirm the role of the cytosine located at the ETS1 and ETS2 motifs, a point mutation of cytosine to thymine was constructed (Fig. 1A). Notably, the ETS1 motif was the critical site for PARP1 transcription only in BRCA1-mutated ovarian cancer cells (Fig. 1Bi-Biv). Recently, a substantial body of evidence suggests that most of the known genes contain specific motifs, such as G-quadruplex in their promoter regions, which can modulate gene transcription by affecting the binding of histone [8] Therefore, CD spectra were used to gain information on whether methylation of the ETS1 motif can influence the structure of the PARP1 promoter. However, our results showed that ETS1 motif methylation may not affect the chemical structure of the core promoter of PARP1 (Fig. 1C).

### Increase of H3K9ac and ETS1 factor enrichment around the hypomethylated ETS1 motif in BRCA1-mutated ovarian cancer

**Figure 2:**
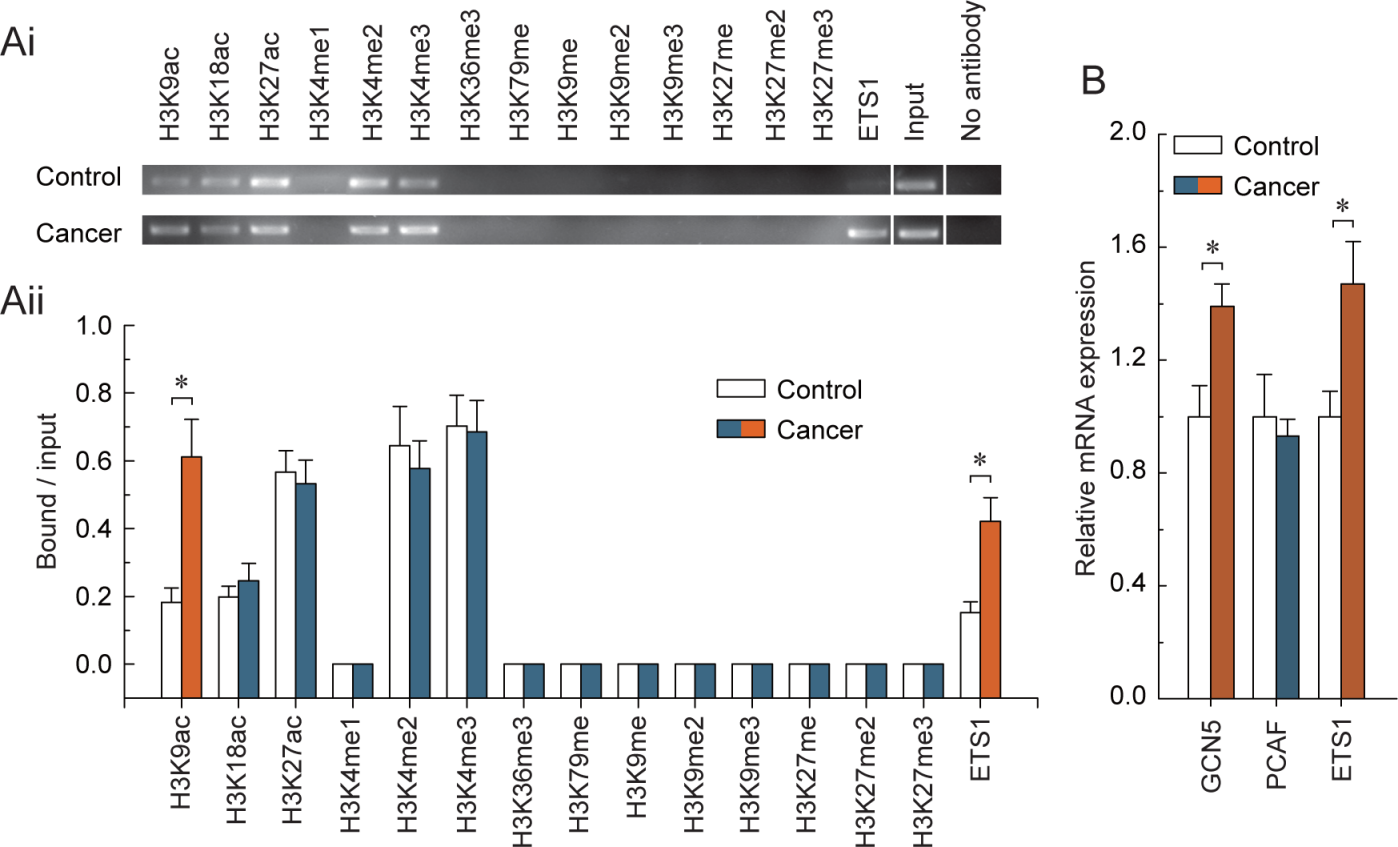
Characteristic histone modification and ETS1 factors enrichment patterns around the hypomethylated ETS1 motif in BRCA1-mutated ovarian cancer (Ai) chromatin immunoprecipitation was performed using antibodies to H3K9ac, H3K18ac, H3K27ac, H3K4me1, H3K4me2, H3K4me3, H3K36me3, H3K79me, H3K9me, H3K9me2, H3K9me3, H3K27me, H3K27me2, and H3K27me3. PCR was performed for regions around the ETS1 motif. A negative control without antibodies was included for comparison. (Aii) representative results of three independent experiments are shown. (B) expression levels of the GCN5, PCAF and ETS1 factors in BRCA1-mutated ovarian cancer. Bar graphs show mean ± SD, *P < 0.05 vs. Control.

To obtain further understanding of the regulatory potential of the crosstalk between DNA methylation and histone modification in controlling PARP1 transcription, we examined the active histone markers H3K9ac, H3K18ac, H3K27ac, H3K4me1, H3K4me2, H3K4me3, H3K36me3 and H3K79me, and the repressive histone markers H3K9me, H3K9me2, H3K9me3, H3K27me, H3K27me2 and H3K27me3 in the core promoter of PARP1, especially at the ETS1 motif; we also focused on the enrichment of the transcription factor ETS1, due to the fact that the hypomethylated ETS1 motif was observed. Chromatin immunoprecipitation analysis indicated that the levels of H3K9ac and ETS1 factors were significantly increased in BRCA1-mutated ovarian cancer tissue (Fig. 2Ai and Aii). The transcription factor ETS1 and chromatin-modifying enzymes that facilitate the creation of histone modification H3K9ac (GCN5 and PCAF) were also analyzed. Although there was no significant change in the expression of PCAF, the expression levels of GCN5 and ETS1 factors were increased in BRCA1-mutated ovarian cancer (Fig. 2B).

### H3K9ac and ETS1 factor present in the ETS1 motif are responsible for the transcriptional regulation of PARP1 in BRCA1-mutated ovarian cancer

**Figure 3:**
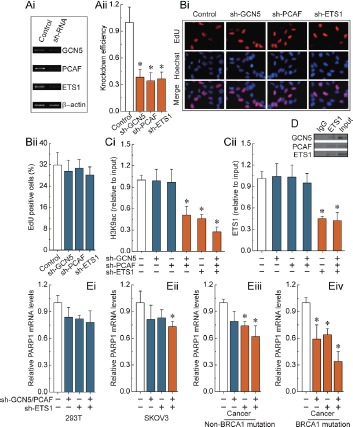
H3K9ac and ETS1-mediated transcriptional regulation of PARP1 (Ai) RT-PCR showing GCN5, PCAF and ETS1 factors levels before and after knockdown by SHRNA, and normalized to β-actin expression. (Aii) representative results of three independent experiments are shown. (Bi) EdU labeling showing proliferation of GCN5, PCAF and ETS1-silenced and control cells. Blue, Hoechst 33342 labeling of cell nuclei; Red, EdU labeling of nuclei of proliferative cells. (Bii) the EdU incorporation rate was expressed as the ratio of EdU positive cells to total Hoechst33342 positive cells. (Ci and Cii) analysis of histone modification H3K9ac and transcription factor ETS1 enrichment around the ETS1 motif after the deletion of GCN5, PCAF or ETS1 factors. (D) The interactions of ETS1 and GCN5 or PCAF were examined by the immunoprecipitation of cell extracts with an antibody to ETS1, and the co-immunoprecipitation of ETS1, GCN5 and PCAF by western blot analysis. Results of Fig.3 A-D were obtained in BRCA1-mutated ovarian cancer cells, and the same results were also obtained in 293T cells, SKOV3 cells, and non-BRCA1 mutated ovarian cancer cells (data not shown). (Ei-Ev) the PARP1 expression levels after deletion of H3K9ac and ETS1 enrichment around the ETS1 motif in 293T cells, SKOV3 cells, and primary non-mutated and BRCA1-mutated ovarian cancer cells. Bar graphs show mean ± SD. *P < 0.05 vs. Control.

We observed that only the combined knockdown of GCN5 and PCAF was able to specifically induce a decrease of H3K9ac around the ETS1 motif (Fig. 3Ci). Interestingly, knockdown of the transcription factor ETS1 showed a similar effect to the combined knockdown of GCN5 and PCAF (Fig. 3Ci). Therefore, an alternative possibility may be that ETS1 factors can recruit GCN5 and PCAF to the ETS1 motif, which is involved in the acetylation of H3K9 (Fig. 3D). As shown in Fig. 3Cii, knockdown of ETS1 factors was an effective way to reduce ETS1 enrichment, but did not change the levels of H3K9ac around the ETS1 motif. Meanwhile, we observed that knockdown of GCN5, PCAF and ETS1 factors (Fig. 2Ai and Aiii) had no detectable effect on the cell morphology and proliferation (Fig. 2Bi and Bii). Notably, we observed that H3K9ac deletion and/or ETS1 enrichment were effective ways to induce a decrease of PARP1 levels in SKOV3 cells and primary non-mutated and BRCA1-mutated ovarian cancer cells (Fig. 3Ei-Eiv). However, the most significant trend was primarily observed in cells originating from BRCA1-mutated ovarian cancer.

### Correlation of ETS1 motif methylation with clinicopathological characteristics in BRCA1-mutated ovarian cancer

As shown in Table 1, the hypomethylated ETS1 motif was associated with high-grade tumor (P = 0.026) and pN1 (P = 0.002). Moreover, ovarian cancer patients with high levels of CA125 (P = 0.190) and high production of ascites (P = 0.077) showed a trend association with ETS1 motif hypomethylation, although this was not statistically significant.

**Table 1:** Association between PARP1 promoter methylation and clinicopathological features of BRCA1-mutated ovarian cancer

	n	M	(%)	UM	(%)	P
Age at diagnosis						0.618
≤ 60 y	40	15	62.50	25	55.56	
> 60 y	29	9	37.50	20	44.44	
Menstrual status						0.761
Premenopausal	15	6	25.00	9	20.00	
Postmenopausal	54	18	75.00	36	80.00	
Histological grade						0.026
I-II	33	16	66.67	17	37.78	
III	36	8	33.33	28	62.22	
pT						0.449
pT1-2	31	9	37.50	22	48.89	
pT3	38	15	62.50	23	51.11	
pN						0.002
pN0	47	22	91.67	25	55.56	
pN1	22	2	8.33	20	44.44	
pM						1.000
pM0	62	22	91.67	40	88.89	
pM1	7	2	8.33	5	11.11	
FIGO stage						0.794
I/II	24	9	37.50	15	33.33	
III/IV	45	15	62.50	30	66.67	
CA125						0.190
≤ 200U/ml	24	11	45.83	13	28.89	
< 200U/ml	45	13	54.17	32	71.11	
Ascites						0.077
≤ 500 ml	35	16	66.67	19	42.22	
> 500 ml	34	8	33.33	26	57.78	
Residual tumor						0.615
≤ 2 cm	31	12	50.00	19	42.22	
> 2 cm	38	12	50.00	26	57.78	
p53 status						0.587
Negative	48	18	75.00	30	66.67	
Positive	21	6	25.00	15	33.33	

FIGO, International Federation of Gynecology and Obstetrics; M, methylated; UM, unmethylated.

### Multivariate and univariate analysis of overall survival for patients with BRCA1-mutated ovarian cancer

**Figure 4:**
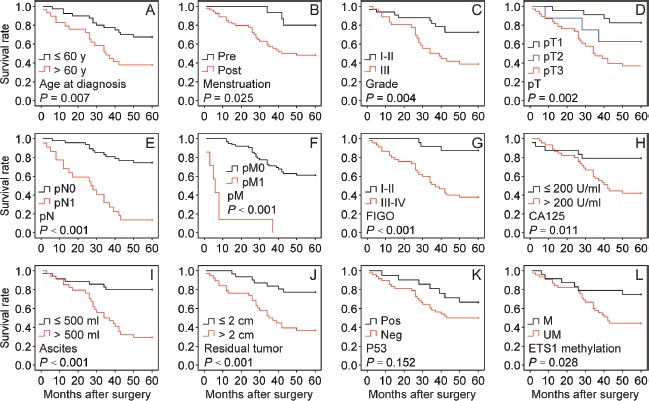
Kaplan-Meier analysis of overall survival for 69 BRCA1-mutated ovarian cancer patients The following variables were analyzed: age at diagnosis, menstruation, grade, pT, pN, pM, FIGO, CA125, ascites, residual tumor, and p53 and ETS1 methylation. Pre, premenopausal; Post, postmenopausal; FIGO, International Federation of Gynecology and Obstetrics; Pos, positive; Neg, negative; M, methylated; UM, unmethylated.

We analyzed the overall survival to assess the prognostic significance. Multivariate Cox regression analysis indicated that histological grade (Table 2, P = 0.001), pT (Table 2, P = 0.018), pM (Table 2, P = 0.001) and p53 status (Table 2, P = 0.007) were independent prognostic factors for predicting the overall survival of BRCA1-mutated ovarian cancer patients. We also performed Kaplan-Meier analysis and log-rank tests for overall survival to define prognostic subgroups. The results revealed that the significant prognostic factors (all P < 0.001) were pN (Fig. 4E), pM (Fig. 4F), FIGO (Fig. 4G), ascites (Fig. 4I) and residual tumor (Fig. 4J). Moreover, patients aged > 60 years (Fig. 4A), those who were postmenopausal (Fig. 4B), and those with Grade III (Fig. 4C), pT3 (Fig. 4D), CA125 (Fig. 4H), and ETS1 motif methylation (Fig. 4L) ovarian cancer showed a significant trend for poor overall survival (all P < 0.05). No significant difference in overall survival was found among patients with different p53 status (Fig. 4K).

**Table 2:** Prognostic factors for overall survival of BRCA1-mutated ovarian cancer patients by multivariate Cox regression analysis

Variable	RR	95% CI	P
Age at diagnosis (> 60 y vs ≤ 60 y)	0.979	0.934-1.026	0.376
Menstrual status (Post vs Pre)	2.938	0.643-13.422	0.164
Histological grade (III vs I-II)	6.536	2.167-19.714	0.001
pT (pT3 vs pT1-2)	3.196	1.222-8.362	0.018
pN (pN1 vs pN0)	2.677	0.703-10.190	0.149
pM (pM1 vs pM0)	11.954	2.847-50.196	0.001
FIGO stage (III-IV vs I-II)	1.287	0.181-9.153	0.801
CA125 (> 200 U/ml vs ≤ 200 U/ml)	0.735	0.221-2.444	0.616
Ascites (> 500 ml vs ≤ 500 ml)	2.221	0.685-7.203	0.184
Residual tumor (> 2cm vs ≤ 2 cm)	1.529	0.579-4.039	0.392
p53 status (Pos vs Neg)	0.255	0.095-0.683	0.007
Methylation (M vs UM)	0.540	0.164-1.778	0.311

Post, postmenopausal; Pre, premenopausal; FIGO, International Federation of Gynecology and Obstetrics; Pos, positive; Neg, negative; M, methylated; UM, unmethylated; RR, relative risk; CI, confidence interval.

## DISCUSSION

Promoter methylation, with concurrent changes in histone modifications, is an epigenetic phenomenon which can affect the conformation of chromatin and the accessibility of DNA for transcription factors [9,10]. ETS is one of the largest transcription factor families and has a highly conserved DNA-binding domain that recognizes a common sequence motif, 5'-(C/A) GGA (A/T) -3' [11], which is widely distributed in the PARP1 promoter [12]. In this study, we report for the first time that (i) ETS1 motif methylation is a key regulatory element for PARP1 transcription, (ii) hypomethylated ETS1-mediated increase of histone modification H3K9ac and transcription factor ETS1 enrichment synergistically activate PARP1 expression in BRCA1-mutated ovarian cancer, and (iii) the deletion of H3K9ac and/or ETS1 enrichment were effective ways to induce a decrease of PARP1 levels in ovarian cancer cells. Moreover, Supplementary Figure 1 shows that BRCA1 status may not directly affect H3K9ac and ETS1 enrichment around the ETS1 motif. Therefore, these results suggest that abnormal hypomethylated ETS1 FIGO, International Federation of Gynecology and Obstetrics; M, methylated; UM, unmethylated.

motif may be the initial factor, but that PARP1 expression is likely to be the result of a complex interaction of multiple factors in a BRCA1-defcient environment, such as increasing the chromatin-modifying enzyme GCN5 levels and transcription factor ETS1 levels. This observation is consistent with previous reports that ETS transcription factors may be key mediators in regulating PARP expression [12]. Furthermore, an increasing amount of evidence suggests that ETS transcription factors are important regulators of the tumorigenic properties of ovarian cancer cells [13] and correlate with poor survival in serous ovarian carcinoma [14].

## METHODS

### Ethics Statement

Investigation has been conducted in accordance with the ethical standards and according to the Declaration of Helsinki and according to national and international guidelines and has been approved by the authors' institutional review board.

### Patient and tissu collection

Serous ovarian cancer patients were enrolled between 2010 and 2012, and all patients gave informed consent. Fresh tumor samples, adjacent normal ovarian tissues, ascites and blood samples were obtained at the time of primary surgery before any chemotherapy or radiotherapy. Hematoxylin and eosin staining of the samples for histopathological diagnosis and grading were determined by three staff pathologists using the World Health Organization criteria. The tumor stages were assessed according to the FIGO and TNM classifications. All patients were screened for BRCA1 mutations by multiplex polymerase chain reaction with complete sequence analysis as previously reported [7]. Their characteristics are given in Table 1.

### Cell culture, lentiviral infection and cell proliferation assay

Primary ovarian cancer cells were obtained from ascites for patients undergoing surgery for ovarian cancer and cultured in RPMI 1640 with 10% fetal bovine serum (Invitrogen, CA USA) as described previously [16]. Human 293T cells and SKOV3 ovarian cancer cells were maintained in DMEM with 10% fetal bovine serum (Invitrogen). Lentiviral vectors expressing short hairpin RNAs (shRNA) against BRCA1 (NM_007299) were obtained from GeneChem Co., Ltd (Shanghai, China), and synthesized as follows: Forward: 5-CCGGAACCTGTCTCCACAAAGTG TGCTCGAGCACACTTTGTGGAGACA GGTTT TTTTG,andReverse:5AATTCAAAAAAACCTGTC TCCA CAAAGTGTGCTCGAGCACACTTTGTG GAGACAGGTT. The non-silencing SIRNA sequence (TTCTCCGAACGTGTCACGT) was used as a negative control. For overexpression of BRCA1, the open reading frame of BRCA1 (NM_007299) was cloned into the lentiviral vector GV287 (Ubi-MCS-3FLAG-SV40-EGFP) (GeneChem). Transfections were performed using the polybrene and enhanced infection solution (GeneChem) according to the manufacturer's recommended protocol. The SHRNA lentiviral particles of GCN5 (sc-37946-V), PCAF (sc-36198-V) and ETS1 (sc-29309-V) were purchased from Santa Cruz Biotechnology (CA, USA). Transfections were performed using the polybrene and enhanced infection solution (GeneChem) according to the manufacturer's recommended protocol. After 48 hours of infection, cell proliferation was determined using the Cell-Light™ EdU Apollo®643 In Vitro Imaging Kit (Ribobio, Guangzhou, China) following the instructions provided by the manufacturer.

### Real-time PCR

Real-time PCR was performed as previously described [7]. The specific primer sequences for real-time PCR are listed in Supplementary Table 1.

### Chromatin immunoprecipitation (CHIP), site-directed mutagenesis, transfection and dual-luciferase reporter assay

CHIP, site-directed mutagenesis, transfection and dual-luciferase reporter assays were performed as previously described [17]. The specific primer sequences for site-directed mutagenesis and CHIP are provided in Supplementary Table 1. The specific antibodies for CHIP are provided in Supplementary Table 2.

### Co-immunoprecipitation (CO-IP) and immunoblotting

CO-IP was performed using an immunoprecipitation kit (Invitrogen) according to the manufacturer's recommended protocol. Then, the cell lysates and immunoprecipitates were analyzed by immunoblotting. The specific antibodies for CO-IP and immunoblotting are provided in Supplementary Table 2.

### Circular dichroism (CD) spectra

The CD spectra were obtained on a Jasco J-810 spectropolarimeter at 25°C using a 0.1 cm path length cell; data were collected with a 2 nm slit width from 350 to 200 nm at 0.5 nm intervals and averaged over three scans. CD experiments were carried out on DNA samples (5 µM) using a buffer containing 0.2 M phosphate buffer (pH 7.0) in the presence of 100 mM Na+ or K+. The DNA samples were annealed by heating to 95°C for 5 min followed by cooling to room temperature over 10 h before analysis. The DNA sequence was 5- CGTGGCGTCGGGCTTC “C (CH3 or non-CH3)” GGAGCTTTGGCGGCAGCTA, and was synthesized by Sangon Biotech Ltd (Shanghai, China).

### Statistical analysis

The association between clinicopathological features and ETS1 motif methylation was determined using the Fisher's exact test. Univariate analysis of survival was performed using the Kaplan-Meier method. Multivariate Cox regression analysis was performed to identify the independent prognostic factors for overall survival. The data are presented as mean ± SD. Statistical differences in the data were evaluated by Student's t test or one-way ANOVA as appropriate, and were considered significant at P < 0.05.

## Supplementary Figures and Tables


