# Supplementary material for: Poly (ADP-ribose) polymerase 1 transcriptional regulation: A novel crosstalk between histone modification H3K9ac and ETS1 motif hypomethylation in BRCA1-mutated ovarian cancer
Source: Oncotarget. 2013 Dec 29;5(1):291–7. doi: 10.18632/oncotarget.1549 (PMC3960209; doi:10.18632/oncotarget.1549)
Supplement: Supplementary file 1 [file oncotarget-05-0291-s001.pdf]

**Supplementary Figure 1.** H3K9ac (A and C) or ETS1 (B and D) enrichment after silencing (A and B) or overexpression (C and D) of BRCA1 in 293T cells, SKOV3 cells, primary non-mutated and BRCA1-mutated ovarian cancer cells.

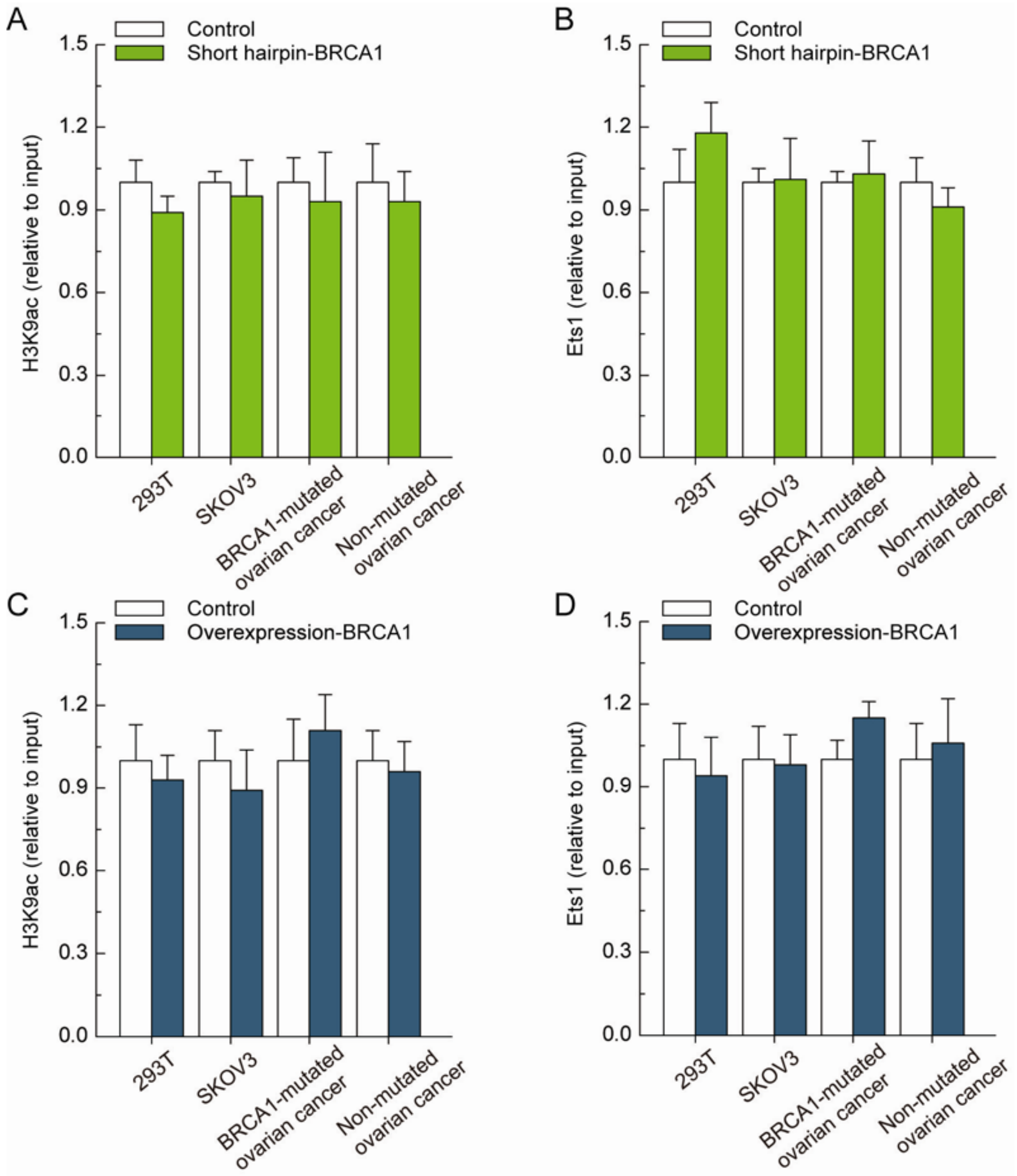

**Supplementary Table 1.** Primers used in this study.

| Gene | Primers | Description |
|---|---|---|
| PARP1-promoter-F | 5'- AATAAAAGGGGGAGGGGTTG | Promoter amplification |
| PARP1-promoter-R | 5'- GAAAGGAGAAGAGAAGAGGC | |
| PARP1-RTP-F | 5'- GAGTCGGCGATCTTGGACC | Real-time PCR for PARP1 |
| PARP1-RTP-R | 5'- TGACCCGAGCATTCCTCG | |
| GAPDH-RTP-F | 5'- AGGTGAAGGTCGGAGTCA | Real-time PCR for GAPDH |
| GAPDH-RTP-R | 5'- GGTCATTGATGGCAACAA | |
| GCN5-SQP | Santa Cruz Biotech<br>GCN5 (h)-PR: sc-37946-PR | Semi-quantitative PCR for<br>GCN5 |
| PCAF-SQP | Santa Cruz Biotech<br>PCAF (h)-PR: sc-36198-PR | Semi-quantitative PCR for<br>PCAF |
| ETS1-SQP | Santa Cruz Biotech<br>ETS-1 (h)-PR: sc-29309-PR | Semi-quantitative PCR for<br>ETS1 |
| PARP1-ChIP-RTP-F | 5'- CAGCGTGTTTCTAGGTCGTG | ChIP for PARP1 (Detection |
| PARP1-ChIP-RTP-R | 5'- GATAGAGCTTATCCGAAGAC | by real-time PCR) |
| PARP1-ChIP-SQP-F | 5'- ATCAGCAATCTATCAGGGAA | ChIP for PARP1 (Detection |
| PARP1-ChIP-SQP-R | 5'- TGCTCTCGCTGCATTTCTTG | by semi-quantitative PCR) |
| PARP1-Mutation1-F | 5'- CCGTGGACGCGGGTTCTGTGGGCGTTCCCGC | A point mutation at the site |
| PARP1-Mutation1-R | 5'- GCGGGAACGCCCACAGAACCCGCGTCCACGG | of -31 (C to T) |
| PARP1-Mutation2-F | 5'- GGCGTCGGGCTTCTGGAGCTTTGGCGGCAGC | A point mutation at the site |
| PARP1-Mutation2-R | 5'- CTGCCGCCAAAGCTCCAGAAGCCCGACGCCA | of +133 (C to T) |

**List of abbreviations used**: F, Forward primer; R, Reverse primer; RTP, Real-time PCR; SQP, Semi-quantitative PCR; ChIP, Chromatin immunoprecipitation.

**Supplementary Table 2.** List of commercial antibodies.

| Antibody | Company (catalog number) | Description |
|---|---|---|
| H3K9Ac | Abcam (ab4441) | ChIP |
| H3K18Ac | Abcam (ab1191) | ChIP |
| H3K27Ac | Millipore (07-360) | ChIP |
| H3K4me1 | Abcam (ab8895) | ChIP |
| H3K4me2 | Abcam (ab7766) | ChIP |
| H3K4me3 | Abcam (ab8580) | ChIP |
| H3K36me3 | Abcam (ab9050) | ChIP |
| H3K79me | Abcam (ab2886) | ChIP |
| H3K9me | Abcam (ab9045) | ChIP |
| H3K9me2 | Millipore (07-441) | ChIP |
| H3K9me3 | Abcam (ab8898) | ChIP |
| H3K27me | Millipore (07-448) | ChIP |
| H3K27me2 | Millipore (07-452) | ChIP |
| H3K27me3 | Millipore (07-449) | ChIP |
| Ets1 | Santa Cruz (sc-350) | ChIP, CO-IP, WB |
| GCN5 | Santa Cruz (sc-20698) | CO-IP, WB |
| PCAF | Santa Cruz (sc-13124) | CO-IP, WB |

**List of abbreviations used**: ChIP, Chromatin immunoprecipitation; CO-IP, Co-immunoprecipitation; WB, Western blotting.
